# Assessing the Structure of the Ways of Coping Questionnaire in Fibromyalgia Patients Using Common Factor Analytic Approaches

**DOI:** 10.1155/2016/7297826

**Published:** 2016-12-13

**Authors:** Charles Van Liew, Maya S. Santoro, Larissa Edwards, Jeremy Kang, Terry A. Cronan

**Affiliations:** ^1^Department of Psychology, San Diego State University, San Diego, CA, USA; ^2^Department of Psychology, Grand Canyon University, Phoenix, AZ, USA; ^3^University of California, San Diego/San Diego State University, Joint Doctoral Program in Clinical Psychology, San Diego, CA, USA

## Abstract

The Ways of Coping Questionnaire (WCQ) is a widely used measure of coping processes. Despite its use in a variety of populations, there has been concern about the stability and structure of the WCQ across different populations. This study examines the factor structure of the WCQ in a large sample of individuals diagnosed with fibromyalgia. The participants were 501 adults (478 women) who were part of a larger intervention study. Participants completed the WCQ at their 6-month assessment. Foundational factoring approaches were performed on the data (i.e., maximum likelihood factoring [MLF], iterative principal factoring [IPF], principal axis factoring (PAF), and principal components factoring [PCF]) with oblique oblimin rotation. Various criteria were evaluated to determine the number of factors to be extracted, including Kaiser's rule, Scree plot visual analysis, 5 and 10% unique variance explained, 70 and 80% communal variance explained, and Horn's parallel analysis (PA). It was concluded that the 4-factor PAF solution was the preferable solution, based on PA extraction and the fact that this solution minimizes nonvocality and multivocality. The present study highlights the need for more research focused on defining the limits of the WCQ and the degree to which population-specific and context-specific subscale adjustments are needed.

## 1. Factor Structure of the Ways of Coping Questionnaire in a Large, Clinical Sample of Fibromyalgia Patients

The Ways of Coping Questionnaire (WCQ) has been a widely used measure of coping processes for the last three decades [1, 2]. The questionnaire was designed to identify the thoughts and actions that individuals use to cope with stress and to discern patterns of coping within specific contexts [3–5]. It has been used to assess coping in response to unique situational stressors, as well as to examine coping within different cultural, occupational, and clinical populations. Despite its use in a variety of populations, including populations with chronic health concerns [3, 6–8], there is no examination of its structure in a sample of individuals with fibromyalgia syndrome (FMS).

### 1.1. The WCQ and Coping

The WCQ is based on the original 68-item Ways of Coping Checklist (WCC), which has a “yes” or “no” response format [9, 10]. The addition of a 4-point Likert response format and the revision of a few of the items to improve clarity were the primary adjustments made in creating the final 66-item version of the WCQ. The use of the WCQ within different populations and across multifarious situational stressors has made evident the complexity and dynamism of the process of coping and attempts to measure this process rigorously [2, 7, 8]. Perhaps the quintessential modern-day approach for assessing the structure of a measure at present is factor analysis [11, 12]. However, factor analyses of the WCQ within and across particular sample types have identified disparate solutions—both in structure and in content—compared to the original 8-factor solution derived by Folkman and Lazarus [10]. These findings may reveal what the genuinely unique coping strategies and structures within different populations and situations are, which may imply that the WCQ should be revised and made population-specific [8].

### 1.2. Original Factor Solution of WCQ

The sample used to obtain the “original” 8-factor solution for the WCQ was comprised of students undergoing examination stress [10]. They used common factor analyses with oblique rotation and found six factors in a sample of undergraduates. Stress and coping were measured at three different time points across the examination process. One of the emotion-focused factors loaded was “rationally” divided into three groups “to provide greater theoretical clarity” ([10], p. 157). After this rational division, eight factors were extracted, and this 8-factor solution was “replicated” in the community sample of 150 married adults [4, 5]. Importantly, Folkman and Lazarus noted neither which specific factor analytic strategies were used nor which factor was divided after solution.

### 1.3. Attempts to Replicate Original Study in Student Samples

In a derivative study that measured coping in undergraduate students who were presented vignettes of stressful encounters, only five of the eight factors were replicated [7]. It is noteworthy that although this solution yielded* fewer factors* than the authors' studies [4, 5], the eigenvalue > 1 rule has been reported to consistently extract too many factors ([13–17]; for a summary, see [11]). The five factors were similar in content to the originally identified domains (see Table 1 for a synopsis of study characteristics). Scherer et al. [7] also did not divide a mathematically extracted factor into three, distinct, “rationally derived” factors. In addition, the stressors presented in the study were hypothetical as opposed to the real stressors measured in the Folkman and Lazarus [10], Folkman et al. [5], and Folkman et al. [4] studies, although these methodological differences may account for the variability in the factors extracted between these studies. Parker et al. [6] measured coping in undergraduate students two days prior to taking their midterm exam (corresponding to Folkman and Lazarus [10]) and failed to find support for the six- and eight-factor oblique and orthogonal models suggested by the student sample [10] and the community sample [4, 5]. These studies started a long history of inconsistency in the structural composition of the WCQ identified via factoring or other dimension reduction techniques (e.g., principal components analysis [PCA]).

### 1.4. Clinical Populations

Beyond these investigations and attempted replications of a general population factor solution of the WCQ, studies of the WCQ factor structure have been conducted among various clinical populations. Surgical, oncological, neurological, rheumatic, and psychiatric patients, as well as caregivers, have been studied. Although some evidence has been found to support the original 8-factor model [1, 18],* most studies* in this domain, as in others, have failed to replicate this solution [19–23]. This may be attributed to the fact that coping is a dynamic process that may be not only different across certain populations but also within particular situations (e.g., compare acute, minor examination stress to enduring, serious medical stress [24–26]), or, again, this could be resultant from variability in study methodologies. Regardless of this, a better understanding of coping in general and in chronically ill populations in particular may be critical for identifying fluctuations in the coping strategies used for managing stress, because the lasting effect of chronic illness likely is different from acute stress (e.g., nonfatal injury, surgery, examination stress, and relationship challenges [27, 28]).

Chronic illnesses defined by a high degree of illness uncertainty (e.g., unknown etiology, prognosis, and cure) may provide a unique perspective on how coping strategies are used, because patients may require more diverse or unique coping skills. To date, only one study has been performed with patients with chronic illnesses that are defined by illness uncertainty (i.e., chronic fatigue syndrome; [18]). Although factor structures of the WCQ have been examined in the general population and various clinical populations—many of whom face unique and notable stressors—to the best of our knowledge, there are no studies that have investigated the factor structure using the WCQ in a sample of patients with fibromyalgia syndrome (FMS), which is defined by illness uncertainty.

### 1.5. Fibromyalgia Syndrome

Fibromyalgia (FMS) is a painful, chronic musculoskeletal disorder defined by stress associated with illness uncertainty. Specifically, FMS has unknown etiology, limited treatment options, and no cure [29, 30]. FMS patients report a plethora of diverse symptoms, including pain, stiffness, fatigue, mood disturbances, memory decline, mental confusion, and sleep disturbance [31–34]. Moreover, individuals with FMS may manage stressors and symptoms in unique ways compared to other chronic pain groups (e.g., significant increases in avoidant strategies; [34]). FMS patients, when compared to other chronic illness populations, have been found to have less health-related hardiness, less resilience to stressors, greater use of passive pain-coping, and worse quality of wellbeing [32, 33, 35–37].

Fibromyalgia patients have been found to have a greater number of comorbid physical and psychological conditions than other chronic pain conditions, as well as experience greater difficulty coping [32, 34]. They also have been found to have lower coping efficacy than other chronic pain populations [32], perhaps in part due to the lack of effective treatments. In a study conducted by Zautra et al. [34], FMS participants reported using significantly more avoidant coping strategies, such as catastrophizing and wishful thinking, than did participants with osteoarthritis, and reported greater average pain and perceived stress. Both groups used similar amounts of active coping; however, among those with chronic pain, strategies such as denial and minimization converge with strategies such as cognitive restructuring and problem-solving as part of “active” coping [34]. Thus, coping in patients with fibromyalgia shares some similarities to other chronic pain populations, yet maintains some differences as well.

The WCQ is commonly used to measure coping in patients with FMS [32, 38–41]. As is evident from the different WCQ structures reported in the literature and the unique, debilitating, and demanding nature of FMS, it is essential to be skeptical of utilizing the same factors found in one group (e.g., cancer patients, student populations) and applying it to another particularized one (i.e., FMS). Therefore, the aim of the present study was both to examine the factor structure of the WCQ in a large sample of individuals diagnosed with FMS and to consider the possibility that the structural diversity of the WCQ across studies is the result of inconsistent statistical methodology.

## 2. Method

### 2.1. Participants

The participants were 501 adults (478 women) who were members of a large Health Maintenance Organization (HMO) and part of a larger intervention study in which no differences between treatment groups were found. Although the sample was largely comprised of females, studies have shown that there is a higher proportion of females comprising samples from rheumatology clinics than in the respective general population [42], and FMS, in particular, disproportionately affects White females [43]. The Institutional Review Boards at both San Diego State University and the Health Maintenance Organization (HMO) reviewed and approved the original study. Data from the 6-month assessment period were used for the present study, because coping was not assessed in the earlier assessment period. The mean age of the participants was 54.27 (SD = 11.17). Participants were primarily White (86.03%) and married (64.67%), and 82.04% had attended some college and 48.10% were employed at least part time (see Table 1 for a summary of demographic characteristics).

### 2.2. Measure

Coping was assessed using the WCQ [44], a self-administered questionnaire consisting of 66 items, grouped into eight subscales. A 4-point rating scale was used; 0 indicated “does not apply and/or not used,” 1 indicated “used somewhat,” 2 indicated “used quite a bit,” and 3 indicated “used a great deal.” There were no instructions to participants about using a particular time frame or to focus on any particular event; thus, participants responded about their coping in general. However, the WCQ was administered as part of an entire test battery that assessed fibromyalgia impact (e.g., the Fibromyalgia Impact Questionnaire, Quality of Well-Being Scale) in a study for which participants were made patently aware of the intention being to study the impact of fibromyalgia.

### 2.3. Procedure

The participants were from a larger study that measured the effects of social support and education on health care use and quality of wellbeing in people with FMS [45]. Participants were recruited through newspaper advertisements, mass mailings to members of a HMO, fliers posted in physicians' offices, and physician referrals. To be eligible, participants had to meet the American College of Rheumatology diagnostic criteria for FMS [46]. Participants completed a series of questionnaires at baseline, 6 months, 1 year, and 18 months following the initial recruitment. The data for the present study were from the 6-month assessment, because it was the only assessment that included a measure of coping. It should be noted that there were no significant intervention effects reported in the study.

### 2.4. Analytic Method

Given the various approaches that have been used to explore the factor structure of the WCQ previously, an extensive consideration of the foundational factoring approaches (i.e., maximum likelihood factoring [MLF], iterative principal factoring [IPF], PAF, and principal components factoring [PCF]) and extraction rules (i.e., eigenvalue > 1, 5% and 10% unique variance explained by a factor, 70% and 80% communal variance explained by extracted factors, Scree plot visual analysis for “elbow” or “joint,” Akaike's and Bayes's Information Criteria [AIC and BIC], and 95th percentile eigenvalue from parallel analysis [PA]) was undertaken. Moreover, for the aforementioned reason and as a result of no preexisting studies having been conducted in FMS samples, the analyses were performed in a purely exploratory fashion. In addition, although factor analysis is, to some extent, inextricably subjective being an unsupervised analytic method [11, 47, 48], evidence from simulation and other studies has suggested that there are some rules which consistently outperform others. One of (if not) the most reliable extraction rules is Horn's [49] PA [11–17, 50–52], which was utilized by our study—apparently for the first time in a study of the WCQ structure in a study conducted in the U.S. The PA was conducted by constructing a random data matrix with vectors of 500 and 66, which were filled with random integers ranging from 1 to 4 using Minitab 16. This matrix was processed with eigendecomposition using the correlation matrix in R 3.0.1, and the 95th percentile eigenvalue for the variable vectors was stored for comparative purposes. Additional analyses were performed in Stata 12.1.

## 3. Results

Initial statistics demonstrated that the data were highly factorable, KMO = .8925; Bartlett's *χ* ^2^  (2145, *N* = 500) = 12355.079, *p* < .001.  Table 2 is a report of the number of factors that would have been retained as a function of both extraction rule and mathematical approach. As is evident from this table, and underwhelming only to those familiar with factoring, even within a single sample there was* great *variability (SD = 8.68) in the solutions acquired as a function of these two variables. Across all rules and methods, the mode number of retained factors was 5 (even when all of the repetitive BIC solutions are counted as only one occurrence of this solution). This number of factors also was retained in the IPF PA, and the loadings for this solution can be found in Table 3. In addition, because 4 factors were retained by two of the PAs—not to mention by the two most common factoring methods, MLF and PAF—the 4-factor solutions from the MLF and PAF are reported in Tables 4 and 5. The tables include loadings only if they were ≥|.30|. If an item had more than one loading of this magnitude, each loading was reported and the item was marked as multivocal. Items that failed to achieve a loading of at least this magnitude on any factor were noted as nonvocal. The 5-factor IPF solution yielded 12 nonvocal items and 3 closely bivocal items; the 4-factor MLF yielded 14 nonvocal items and 1 closely bivocal item; and the 4-factor PAF yielded 10 nonvocal items and 1 closely bivocal item (see Tables 4 [Table tab5]–6 for details). Beyond reviewing vocality, solutions were considered for their interpretability. Based on these criteria, the 4-factor PAF solution was selected as the preferable solution, because it minimizes nonvocality and multivocality and yields a highly interpretable solution. The four extracted factors—in order of extraction—were labeled by the authors as detachment, deliberative coping, emotional coping, and wishful coping (see Table 7 for interfactor correlations).

## 4. Discussion

In 1985, Folkman and Lazarus published their factor structure for the WCQ. Their initial factor analysis was performed on a sample of undergraduate students stressed by exams. The results yielded eight coping subscales (after rationally subdividing one mathematically extracted factor into three factors). In 1986, they replicated their factor structure on a community sample and again found the same eight coping subscales. Yet, Folkman [53] recommended testing the factor structure across populations, because they believed it was likely to change. Although some researchers followed this suggestion (e.g., [21, 54, 55]), many others have simply used the factor structure found in the original study.

Studies have demonstrated that patients with chronic pain conditions, including FMS, used a variety of coping strategies, including active and passive forms of coping [34]. Past research has further demonstrated a variety of common coping strategies in the FMS population that map onto the WCQ factors, including reinterpreting pain symptoms, ignoring pain symptoms, distraction, use of coping self-talk, prayer/hope, seeking support, and increased activity level (e.g., [36, 56]). Studies examining coping in FMS patients use measures that were not developed or normed in that patient population. The present study attempts to explore factor structures of coping on a widely used measure among a large sample of FMS patients to further research in this area.

In the present study, the factor structure of the WCQ was tested on a group of 501 participants with fibromyalgia who were predominantly (95%) women. The solution deemed most reasonable (a PAF, oblique oblimin rotated solution selected with PA) included four factors. We named the four factors: detachment, deliberative coping, emotional coping, and wishful coping, respectively. The number of factors in our solution was half of those found of Folkman and Lazarus [10], and ten of the original items from the WOC questionnaire were nonvocal and were removed in the 4-factor solution. The number of items in our four-factor subscales was greater than that found by Folkman and Lazarus [10]. This new factor structure may indicate that the coping strategies used by people with FMS may differ qualitatively from those of college students undergoing the stress of exams—as in the original study. In addition, because the majority of the items were retained in our factor solution, it may indicate that the WCQ captures similar coping experiences of people across populations, demonstrating the robustness of the scale.

Other researchers who have examined the factor structure of the WOC questionnaire have reported between three- [8, 19, 22, 55] and eight-factor solutions [1, 18, 57]. These studies were conducted on a variety of clinical populations (e.g., adults with spinal cord injuries, multiple sclerosis, adults with chronic fatigue syndrome and their caregivers, female breast cancer patients, and adult surgery patients), nonclinical populations (e.g., teachers, health care workers, and students), and in multiple countries (e.g., United States, China, Iran, Turkey, Sweden, Canada, and Taiwan). The differences in factor structures may be attributed to differences in coping strategies employed across populations, cultures, conditions, and other such factors. Potential explanations for differences in the factor structure also could be attributed to differences in factor analytic strategies used and modifications made to the questionnaires across studies. The variability in factor analytic approaches can be seen in Table 1, which shows that sample sizes, mathematical methods, and extraction rules have varied substantially across studies of this measure. Moreover, some researchers have modified the WOC questionnaire in terms of the number of items, the response format [20], and item wording [18, 19].

In spite of methodological differences, it is likely that at least some of the differences in factor structures across studies can be attributed to* actual *group differences in coping experiences (e.g., population level difference, cultural differences). Coping is a complex phenomenon impacted by a variety of personal and situational factors [58]. For instance, the experience of people with fibromyalgia coping with illness uncertainty and chronic symptoms (e.g., pain, fatigue) may be qualitatively and quantitatively different from the experience of coping with acute stresses, such as undergoing exams in an academic setting. It is easy to envision the differences in coping approaches that may exist between such seemingly dissimilar stressors. However, there may also be coping differences across illness conditions because of the unique set of stressors and demands of different illnesses. Our four factors differed from those found in other chronic conditions, including breast cancer [19], gynecological cancer [59], and multiple sclerosis or spinal cord injuries [22]. These differences may also be attributed to the different types of treatments and their differing levels of effectiveness. For example, there is no cure for FMS, and the treatments prescribed vary depending on type of provider, the length of illness, and other comorbid conditions (e.g., [60, 61]).

In the context of the present study, it is important to note that participants were not told to consider coping with fibromyalgia explicitly within the instructions of the WCQ. However, given the participants' awareness of the intention of the study and the inclusion of the WCQ in a battery of tests which assessed fibromyalgia impact, it seems reasonable to infer that participants were considering their “general coping” with fibromyalgia (as opposed to specific moments or instances of coping with fibromyalgia) based on the instructions of the WCQ. Yet, it must be acknowledged that there may be some variability among responses of participants with respect to whether they were considering global, general coping or illness-related general coping. The consistency of the present study with past research in FMS disease-specific coping suggests that the assumption of the illness-related general coping is reasonable and tenable.

It is possible that this factor structure might generalize to patients coping with other chronic pain conditions, because across conditions patients must similarly contend with ongoing pain, which presents a pervasive stressor leading to physical and psychosocial impairments. It is also likely that this factor structure may not generalize to some other chronic pain conditions, especially those that are more predictable in nature or whose treatment options are more effective. The demands of FMS likely differ from many other conditions for which treatments are less variable and more effective, and less illness uncertainty exists. Reich et al. [62] found that illness uncertainty moderated the effect of daily stressors in predicting affective state in a longitudinal study of FMS patients. Thus, fibromyalgia patients may face unique coping experiences related to illness uncertainty, chronic symptoms, and treatment challenges, and these experiences likely require particularized coping approaches.

As an example of similarities and differences across chronic pain populations, Newth and DeLongis [23] conducted a similar study among patients with rheumatoid arthritis (RA). The factor solution of the WCQ found in the current study has similarities and differences when compared to those found in other pain populations. One problem in comparing factor structures includes variation in how factor names are conceptualized and the statistical procedures and protocols used to determine factor structure. Newth and DeLongis [23] reported a four-factor solution; however, they did not provide information regarding the statistical procedures and protocols used. Their factors appear similar to those found in the current study, except for some differences in what was conceptualized as* detachment* in the current study and what Newth and DeLongis termed* cognitive reframing* and* distancing*. Newth and DeLongis also had far fewer items, 18 in all, included in their factor solution compared to the present study with 56 vocal items. In particular, two items regarding perspective taking and comparison to others, namely, the items* I thought about someone I know who is in a worse situation *and* I realized how, in some ways, I'm more fortunate than others*, failed to load in the current study but did load in the Newth and DeLongis study. Also notably different is the decreased number of* wishful thinking* items that loaded in the present study and not in the Newth and DeLongis study. Therefore, it appears that the fibromyalgia sample endorsed greater amounts of avoidant coping strategies than the rheumatoid arthritis sample. This is consistent with the coping patterns found in FMS patients from past studies (e.g., [34]).

It is interesting to note that the 10 nonvocal items in the present study included primarily proactive strategies (e.g., taking a big chance, analyzing problems, doing something even if it was not expected to work, seeking professional help, and getting a responsible party to change his or her mind). Beyond this, the following items did not load: preparing for the worst, praying, leaving things open, accepting the next best thing, and sleeping more. Given the sleep disturbance that is common among FMS patients [31], it is not surprising that “sleeping more” might not be a viable coping option. The feelings of helplessness that FMS can elicit may be related to decreased use of some of these strategies as well. Consistent with this notion, Reich et al. [62] found that illness uncertainty impacted daily coping in a sample of FMS patients. This is consistent with many of these additional nonvocal items and the fact that “wishful coping” was the factor that accounted for the least variance of those retained.

Furthermore, given that there are various factors that affect coping (e.g., age, gender) and that participants in the present study were asked to report general coping processes, it is possible that unique structures could be found within other pain populations that differ systematically on other important determinants of coping. According to the authors [44], the WCQ was designed to be answered with a specific stressful encounter in mind; however, there was no standardized method developed for obtaining that information. Hence, several studies have included the questionnaire to assess general coping strategies rather than as a method of assessing coping in reference to a particular event [4, 5, 9, 18, 63]. Other studies have included the measure but do not include details of the instructions to the participants when answering the questionnaire [64, 65]. It would be beneficial to explore the coping structures that emerge when responses to specific stressors are elicited among those with FMS samples. Although there is variability in the solutions obtained from populations, it is important to note that the factor solutions we found for the WCQ are similar to some of the factors others have found. For instance, Hwang et al., [55] who included four samples of health workers and teachers, found a seven-factor solution. Four of their seven factors had significant overlap with our four subscales. Chan [54] also found a four-factor solution with participants who were adolescent students and teachers. Chan named his factors similarly to those in the present study. Specifically, he named them problem-solving, resigned distancing, seeking social support and ventilation, and passive wishful thinking. All factors had multiple items that overlapped with our four factors. Across studies, many of the original WCQ items were retained, suggesting that the WCQ is relatively robust at the item level. However, there is variability in the number and the composition of factors found, as well as the number and content of items that load onto each factor. Thus, there are robust components to the WCQ and also some characteristics that make it unique for each of the samples tested.

The WCQ subscales are designed to provide information about areas of coping strengths and weaknesses, which may provide important information for clinical and research assessment and intervention. As such, it is crucial that accurate subscales are developed and used to assess coping for different populations. For future researchers using the WCQ, findings from the present study highlight the potential benefit of conducting factor analysis within the sample of interest and using the resulting factor structure to measure coping rather than relying on the original eight-factor solution. Furthermore, in defining these structures, it behooves researchers to name their factor solutions using nonjudgmental labels, so that there is an appreciation that the quality of a particular coping strategy may be dependent on how and when it is employed. Using nonjudgmental labels allows for the same coping strategy to be both functional and dysfunctional depending on these conditions.

The present study highlights the need for more research focused on defining the limits of the WCQ and the degree to which population-specific and context-specific subscale adjustments are needed. Within the FMS population, a better understanding is needed to determine whether this four-factor solution can be applied to all FMS patients or whether it is applicable to specific subtypes of patients. FMS is a heterogeneous population in terms of symptom presentation, treatment response, and degree of coping effectiveness [60, 66–68]. Despite the growing appreciation of FMS as a heterogeneous patient population, FMS patients are typically assessed and treated as a homogeneous population. It is critical that future research efforts focus on developing a comprehensive understanding of the FMS experience, particularly in the area of coping.

In moving forward, it is important that researchers are clear and consistent with respect to the methods employed. Our solution, like those from previous studies, is not a “one-size-fits-all” solution. Idiosyncrasies of our sample include coming from a small geographic region, being primarily White and female, and being well educated and well-to-do (see Table 2). Therefore, it is important for future researchers to continue to examine different factor solutions that may occur as a function of gender, race, culture, age, experiential, and contextual factors within different samples and to attempt to replicate factor solutions using confirmatory approaches which align closely with previous studies (e.g., population type, sample size, extraction rule). This will help to determine the degree to which variability in solutions is the result of genuine population differences or methodological and analytic discrepancies. As has been known for some time, PA is a superlative extraction rule [11–17, 50–52]. Although Horn incepted it in 1965, its use has been lacking throughout factor analytic studies in the behavioral sciences. Our study, along with the two most recent studies of the WCQ (both of which were conducted in non-U.S. samples), is among the first to use PA as the rule for extraction. The use of oblique rotation seems to be indicated strongly based on the conceptual nature of coping (i.e., strategies are likely interrelated, not orthogonal [see Table 7 for the interfactor correlations from the present study for the 4-factor PAF approach]), as others have noted previously with respect to the WCQ [5, 10, 20, 65]. A final important step to consider in determining robust solutions that are population-appropriate is moving toward a supervised version of dimension reduction, such as partial least squares or principal components regressions [47]. These techniques identify solutions that are useful in specific contexts and populations as predictors of important outcomes and are able to be evaluated and replicated more objectively within those specific domains and for those specific outcomes. This would be useful in establishing the predictive validity of obtained factor solutions. For example, the solutions could be developed as useful predictors of important outcomes within the settings from which they are derived (e.g., recovery from surgery, quality of wellbeing, psychological health, and academic success). Such models could prove to make the WCQ an even more useful instrument than it currently is.

## Figures and Tables

**Table 1:** Summary of previous factor analytic studies of the WOC.

Authors	Year	Population	Stressor	*n*	Approach	Rotation	Extraction rule	Factors	Names of factors
Folkman & Lazarus	1985	Californian undergraduate students	In situ: exams	324 (108 independent)	“Common factor analyses” (NOS)	Oblique	Interpretability	6 (8)^*∗*^	Confrontive coping Distancing Self-controlling Seeking social support Accepting responsibility Escape-avoidance Planful problem-solving Positive reappraisal

Vitaliano et al.	1985	Psychiatric outpatients; Alzheimer's disease spousal caregivers; medical students°	In situ: anger; caregiving; occupation-education	83; 62; 425	PCA	Orthogonal (varimax)	Eigenvalue > 1 Interpretability	6(5)^∙^	Problem-focused coping Blamed self Wishful thinking Seeking social support Avoidance

Folkman et al.	1986	White, Southern Californian married adults (women aged 35–45 years)	In situ: child-rearing	150	Alpha factoring; PAF (8 factors preselected)	Oblique	Interpretability	8	Confrontive coping Distancing Self-controlling Seeking social support Accepting responsibility Escape-avoidance Planful problem-solving Positive reappraisal

Aldwin & Revenson	1987	Southern Californian adults	In situ: recalling any recent stressor	291	PAF	Orthogonal (varimax) Oblique	Interpretability “Clearest and most interpretable results on both conceptual and empirical grounds” (p. 340)	8	Escapism Cautiousness Instrumental action Minimization Support mobilization Self-blame Negotiation Seeking meaning

Scherer et al.	1988	Southern U.S. undergraduate students	Ersatz: stressful events vignettes	491	Adopted Folkman and Lazarus [10] approach (NOS)	Oblique	Eigenvalue > 1	5	Problem-focused detachment Wishful thinking Seeking social support Focusing on the positive

Parker et al.	1993	Ontarian undergraduate students	In situ: exams	Study 1 (Derivation): 530 Study 2 (Replication): 392	PAF	Oblique Orthogonal	Eigenvalue > 1 Scree plot visual analysis	4	Confrontive/seeking social support Problem-focused Denial Distancing/avoiding [Not replicated]

Mishel & Sorenson	1993	Female gynecological cancer patients	In situ: newly diagnosed, undergoing treatment	231	PCA	Orthogonal (varimax)	Not specified	7	Bargaining Focusing on the positive Social support Concentrated efforts Wishful thinking Detachment Acceptance

Chan	1994	Chinese students and teachers	In situ: Events of daily living	657	MLF	Orthogonal (varimax)	Scree plot visual analysis TLI Interpretability 5% unique variance	4	Rational problem-solving Resigned distancing Seeking support and ventilation Passive wishful thinking

Wineman et al.	1994	Adults with spinal cord injury or multiple sclerosis	In situ: disease-related	655	EFA	Orthogonal (quartimax)	Eigenvalue > 1 Scree plot visual analysis	3	Cognitive reframing Emotional respite Direct assistance

Smyth & Yarandi	1996	Working African-American women	In situ: work stressors	656	PFA	Oblique	Scree plot visual analysis	3	Active coping Avoidant coping Minimizing the situation

Ax	1999	Adults with chronic fatigue syndrome, myalgic encephalomyelitis, or postviral fatigue; adult caregivers	In situ: disease-related; caregiving	155; 95	PCA	Orthogonal (varimax) and convergence	Scree plot visual analysis	8	Confrontive coping Distancing Self-controlling Seeking social support Accepting responsibility Escape-avoidance Planful problem-solving Positive reappraisal

Sørlie & Sexton^†^	2001	Adult surgery patients; adult surgery patients	In situ: surgery	555; 482	PAF	Orthogonal (varimax)	Eigenvalue > 1 Scree plot visual analysis	5	Wishful thinking Goal-oriented Seeking support Thinking it over Avoidance

Rosberger et al.	2002	Female breast cancer patients	In situ: newly diagnosed	156	FA (NOS)	Oblique	Scree plot visual analysis	3	Positive problem-solving Escape/avoidance Seeking social support

Hwang et al.	2002	U.S. healthcare workers; Chinese healthcare workers; Chinese teachers; Taiwanese teachers	In situ: work stressors	682; 396; 372; 364	PCA	Orthogonal (varimax)	Eigenvalue > 1	3^§^	Planning Positive reappraisal Distancing

Lundqvist & Ahlström	2006	Swedish adults with neurological diseases; Swedish next-of-kin; Swedish students	In situ: disease-related; caregiving; education	219; 77; 214	Confirmatory MLF	Oblique	RMSEA GFI AGFI CFI IFI	8	Confrontive coping Distancing Self-controlling Seeking social support Accepting responsibility Escape-avoidance Planful problem-solving Positive reappraisal

Senol-Durak et al.	2011	Turkish undergraduate students; Turkish adults	Not specified: administered in classroom, work, and home environments	Study 1: 472 Study 2: 485 Study 3: 416	PCA	Oblique	Eigenvalue > 1 PA MAP	20 8 7^‡^	Planful problem-solving Keep to self Seeking social support Accept responsibility Escape-avoidance Refuge in supernatural forces Refuge in fate

Padyab et al.	2012	Iranian adults	In situ: most recent stressful event	739	PCA	Oblique	PA	7	Confrontive coping Distancing Self-control Seeking social support Escape-avoidance Planful problem-solving Positive reappraisal

*Note*. PCA = principal components analysis; PAF = principal axis factoring; MLF = maximum likelihood factoring; PA = parallel analysis; MAP = minimum average partial; TLI = Tucker-Lewis Index; RMSEA = root mean squared error of approximation; GFI = Good of Fit Index; AGFI = Adjusted Goodness of Fit Index; CFI = Comparative Fit Index; IFI = Incremental Fit Index; NOS = not otherwise specified. ^*∗*^Six factors were extracted mathematically, but one factor was divided (based on rationale) into three unique factors. °The medical student sample was used as sample for the primary analysis to determine the number of factors. ^∙^Six factors were extracted mathematically, but two factors were combined (based on rationale and the low numbers of items that loaded onto the factors). ^§^Three factors were chosen, because these were the only ones that replicated across each of the four samples. ^†^A 5-point Likert-type scale supplanted the original 4-point scale in this study. ^‡^This was the apparently preferred solution to the authors.

**Table 2:** Demographic variables.

Variable	Mean (SD)/percentages (*N*)
*Age*	54.31 (11.20)
*Gender*	
Male	4.59 (23)
Female	95.41 (478)
*Ethnicity *	
White	86.03 (431)
Non-White	13.57 (68)
Declined to state	.4 (2)
*Marital status*	
Not married	35.33 (177)
Married	64.67 (324)
*Employment*	
Not working	51.90 (260)
Working	48.10 (241)
*Education*	
High school or less	17.76 (89)
At least some college	82.04 (411)
Declined to state	.20 (1)
*Income*	
Below $10,000	5.39 (27)
$10,001–$20,000	10.38 (52)
$20,001–$30,000	15.77 (79)
$30,001–$40,000	19.96 (100)
$40,001–$50,000	14.97 (75)
$50,001–$60,000	9.58 (48)
$60,001–$70,000	7.19 (36)
Above $70,000	13.37 (67)
Decline to state	3.39 (17)
*Length of symptoms (yrs)*	6.02 (8.4)

**Table 3:** Factors extracted by mathematical method and factoring rule.

Rule	Approach			
	MLF	IPF	PAF	PCF
Eigenvalue > 1	7	9	7	16
5% unique variance	5	3	5	3
10% unique variance	3	2	3	1
70% communal variance	5	14	4	23^‡^
80% communal variance	7	21	6	32^‡^
AIC	26^‡^	—	—	—
BIC	5	—	—	—
Scree^**∗**^	5	6	5	5
≥95% PA eigenvalue^†^	4	5	4	6

*Note. *AIC = Akaike's Information Criteria; BIC = Bayes's Information Criteria; PA = parallel analysis; MLF = maximum likelihood factoring; IPF = iterative principal factoring; PAF = principal axis factoring; PCF = principal components factoring. ^**∗**^Scree estimates include the elbow in the number of factors. ^†^PA 95% eigenvalue = 1.6276. ^‡^Heywood solution.

**Table 4:** Five-factor IPF solution.

Item	Factor 1	Factor 2	Factor 3	Factor 4	Factor 5
*Just concentrated on what I had to do next – the next step.*	*Nonvocal*				
I tried to analyze the problem in order to understand it better.		.35			
*Turned to work or substitute activity to take my mind off things.*	*Nonvocal*				
I felt that time would make a difference – the only thing to do was to wait.	.32				
Bargained or compromised to get something positive from the situation.			.32		
*I did something which I didn't think would work, but at least I was doing something.*	*Nonvocal*				
**Tried to get the person responsible to change his or her mind.**		**.33**	**.38**		
Talked to someone to find out more about the situation.		.39			
Criticized or lectured myself.				.31	
Tried not to burn my bridges, but leave things open somewhat.		.35			
Hoped a miracle would happen.				.57	
*Went along with fate; sometimes I just have bad luck.*	*Nonvocal*				
Went on as if nothing had happened.	.47				
I tried to keep my feelings to myself.					−.54
Looked for the silver lining, so to speak; tried to look on the bright side of things.	.78				
*Slept more than usual.*	*Nonvocal*				
I expressed anger to the person(s) who caused the problem.			.57		
Accepted sympathy and understanding from someone.					.34
I told myself things that helped me to feel better.		.41			
I was inspired to do something creative.	.43				
Tried to forget the whole thing.	.48				
*I got professional help.*	*Nonvocal*				
Changed or grew as a person in a good way.	.64				
*I waited to see what would happen before doing anything.*	*Nonvocal*				
I apologized or did something to make up.			.39		
I made a plan of action and followed it.		.53			
*I accepted the next best thing to what I wanted.*	*Nonvocal*				
**I let my feelings out somehow.**			**.37**		**.33**
Realized I brought the problem on myself.			.53		
I came out of the experience better than when I went in.	.64				
**Talked to someone who could do something concrete about the problem.**		**.40**			**.45**
*Got away from it for a while; tried to rest or take a vacation.*	*Nonvocal*				
Tried to make myself feel better by eating, drinking, smoking, using drugs or			.39		
Took a big chance or did something very risky.			.32		
I tried not to act too hastily or follow my first hunch.		.36			
Found new faith.	.35				
Maintained my pride and kept a stiff upper lip.	.58				
Rediscovered what is important in life.	.69				
Changed something so things would turn out all right.		.44			
Avoided being with people in general.				.35	
Didn't let it get to me; refused to think too much about it.	.56				
I asked a relative or friend I respected for advice.					.51
Kept others from knowing how bad things were.					−.42
Made light of the situation; refused to get too serious about it.	.57				
Talked to someone about how I was feeling.					.57
Stood my ground and fought for what I wanted.			.32		
Took it out on other people.			.47		
Drew on my past experiences; I was in a similar situation before.		.49			
I knew what had to be done, so I doubled my efforts to make things work.		.48			
*Refused to believe that it had happened.*	*Nonvocal*				
I made a promise to myself that things would be different next time.		.41			
Came up with a couple of different solutions to the problem.		.74			
Accepted it, since nothing could be done.	.38				
I tried to keep my feelings from interfering with other things too much.		.31			
Wished that I could change what had happened or how I felt.				.69	
*I changed something about myself.*	*Nonvocal*				
I daydreamed or imagined a better time or place than the one I was in.				.60	
Wished that the situation would go away or somehow be over with.				.78	
Had fantasies or wishes about how things might turn out.				.61	
I prayed.	.41				
*I prepared myself for the worst.*	*Nonvocal*				
I went over in my mind what I would say or do.		.56			
I thought about how a person I admire would handle this situation and used that		.44			
I tried to see things from the other person's point of view.		.37			
I reminded myself how much worse things could be.	.44				
I jogged or exercised.	.34				

*Note.* Items with loadings on more than one factor ≥ .30 but where the loadings are ≥ .10 different in absolute magnitude are not considered bivocal, but all loadings ≥ .30 are reported in table. Bivocal items are in bold font. Nonvocal items are in italic font.

**Table 5 tab5:** Four-factor MLF solution.

Item	Factor 1	Factor 2	Factor 3	Factor 4
Just concentrated on what I had to do next – the next step.	.37			
*I tried to analyze the problem in order to understand it better.*	*Nonvocal*			
Turned to work or substitute activity to take my mind off things.	.37			
*I felt that time would make a difference – the only thing to do was to wait.*	*Nonvocal*			
Bargained or compromised to get something positive from the situation.	.35			
*I did something which I didn't think would work, but at least I was doing something.*	*Nonvocal*			
*Tried to get the person responsible to change his or her mind.*	*Nonvocal*			
Talked to someone to find out more about the situation.		.45		
Criticized or lectured myself.				.40
*Tried not to burn my bridges, but leave things open somewhat.*	*Nonvocal*			
Hoped a miracle would happen.				.50
Went along with fate; sometimes I just have bad luck.				.30
Went on as if nothing had happened.	.57			
I tried to keep my feelings to myself.	.51			
Looked for the silver lining, so to speak; tried to look on the bright side of things.	.67			
*Slept more than usual.*	*Nonvocal*			
I expressed anger to the person(s) who caused the problem.			.51	
Accepted sympathy and understanding from someone.			.38	
I told myself things that helped me to feel better.		.53		
I was inspired to do something creative.	.42			
Tried to forget the whole thing.	.49			
*I got professional help.*	*Nonvocal*			
Changed or grew as a person in a good way.	.57			
I waited to see what would happen before doing anything.			.33	
I apologized or did something to make up.			.43	
I made a plan of action and followed it.		.64		
*I accepted the next best thing to what I wanted.*	*Nonvocal*			
I let my feelings out somehow.			.47	
Realized I brought the problem on myself.			.58	
I came out of the experience better than when I went in.	.56		.34	
Talked to someone who could do something concrete about the problem.		.63		
Got away from it for a while; tried to rest or take a vacation.		.38		
Tried to make myself feel better by eating, drinking, smoking, using drugs or			.37	
*Took a big chance or did something very risky.*	*Nonvocal*			
I tried not to act too hastily or follow my first hunch.		.37		
Found new faith.		.30		
Maintained my pride and kept a stiff upper lip.	.69			
Rediscovered what is important in life.	.58			
Changed something so things would turn out all right.		.49		
Avoided being with people in general.				.40
Didn't let it get to me; refused to think too much about it.	.59			
I asked a relative or friend I respected for advice.		.47	.33	
Kept others from knowing how bad things were.	.45			.31
Made light of the situation; refused to get too serious about it.	.67			
Talked to someone about how I was feeling.		.54		
*Stood my ground and fought for what I wanted.*	*Nonvocal*			
Took it out on other people.			.43	
Drew on my past experiences; I was in a similar situation before.		.42		
**I knew what had to be done, so I doubled my efforts to make things work.**	**.39**	**.40**		
*Refused to believe that it had happened.*	*Nonvocal*			
I made a promise to myself that things would be different next time.		.38		
Came up with a couple of different solutions to the problem.		.70		
Accepted it, since nothing could be done.	.37			
I tried to keep my feelings from interfering with other things too much.	.38			
Wished that I could change what had happened or how I felt.				.73
I changed something about myself.		.34		
I daydreamed or imagined a better time or place than the one I was in.				.57
Wished that the situation would go away or somehow be over with.				.76
Had fantasies or wishes about how things might turn out.				.58
*I prayed.*	*Nonvocal*			
*I prepared myself for the worst.*	*Nonvocal*			
I went over in my mind what I would say or do.		.41		
I thought about how a person I admire would handle this situation and used that		.54		
I tried to see things from the other person's point of view.		.45		
I reminded myself how much worse things could be.	.39			
*I jogged or exercised.*	*Nonvocal*			

*Note.* Items with loadings on more than one factor ≥ .30 but where the loadings are ≥ .10 different in absolute magnitude are not considered bivocal, but all loadings ≥ .30 are reported in table. Bivocal items are in bold font. Nonvocal items are in italic font.

**Table 6:** Four-factor PAF solution.

Item	Factor 1	Factor 2	Factor 3	Factor 4
Just concentrated on what I had to do next – the next step.	.39			
*I tried to analyze the problem in order to understand it better.*	*Nonvocal*			
Turned to work or substitute activity to take my mind off things.	.39			
I felt that time would make a difference – the only thing to do was to wait.	.31			
Bargained or compromised to get something positive from the situation.	.36			
*I did something which I didn't think would work, but at least I was doing something.*	*Nonvocal*			
*Tried to get the person responsible to change his or her mind.*	*Nonvocal*			
Talked to someone to find out more about the situation.		.44		
Criticized or lectured myself.				.42
*Tried not to burn my bridges, but leave things open somewhat.*	*Nonvocal*			
Hoped a miracle would happen.				.52
Went along with fate; sometimes I just have bad luck.				.33
Went on as if nothing had happened.	.59			
I tried to keep my feelings to myself.	.53			
Looked for the silver lining, so to speak; tried to look on the bright side of things.	.68			
*Slept more than usual.*	*Nonvocal*			
I expressed anger to the person(s) who caused the problem.			.54	
Accepted sympathy and understanding from someone.			.39	
I told myself things that helped me to feel better.		.54		
I was inspired to do something creative.	.43			
Tried to forget the whole thing.	.51			
*I got professional help.*	*Nonvocal*			
Changed or grew as a person in a good way.	.57			
I waited to see what would happen before doing anything.			.31	
I apologized or did something to make up.			.42	
I made a plan of action and followed it.		.62		
*I accepted the next best thing to what I wanted.*	*Nonvocal*			
I let my feelings out somehow.			.49	
Realized I brought the problem on myself.			.56	
I came out of the experience better than when I went in.	.56		.33	
Talked to someone who could do something concrete about the problem.		.63		
Got away from it for a while; tried to rest or take a vacation.		.40		
Tried to make myself feel better by eating, drinking, smoking, using drugs or			.35	
*Took a big chance or did something very risky.*	*Nonvocal*			
I tried not to act too hastily or follow my first hunch.		.37		
Found new faith.		.32		
Maintained my pride and kept a stiff upper lip.	.69			
Rediscovered what is important in life.	.59			
Changed something so things would turn out all right.		.50		
Avoided being with people in general.				.42
Didn't let it get to me; refused to think too much about it.	.60			
I asked a relative or friend I respected for advice.		.47	.34	
Kept others from knowing how bad things were.	.46			.32
Made light of the situation; refused to get too serious about it.	.68			
Talked to someone about how I was feeling.		.54		
Stood my ground and fought for what I wanted.			.34	
Took it out on other people.			.43	
Drew on my past experiences; I was in a similar situation before.		.41		
**I knew what had to be done, so I doubled my efforts to make things work.**	**.38**	**.40**		
Refused to believe that it had happened.				.31
**I made a promise to myself that things would be different next time.**		**.39**		**.32**
Came up with a couple of different solutions to the problem.		.70		
Accepted it, since nothing could be done.	.38			
I tried to keep my feelings from interfering with other things too much.	.39			
Wished that I could change what had happened or how I felt.				.71
I changed something about myself.		.34		
I daydreamed or imagined a better time or place than the one I was in.				.60
Wished that the situation would go away or somehow be over with.				.75
Had fantasies or wishes about how things might turn out.				.62
*I prayed.*	*Nonvocal*			
*I prepared myself for the worst.*	*Nonvocal*			
I went over in my mind what I would say or do.		.43		
I thought about how a person I admire would handle this situation and used that		.56		
I tried to see things from the other person's point of view.		.47		
I reminded myself how much worse things could be.	.39			
I jogged or exercised.	.31			

*Note.* Items with loadings on more than one factor ≥ .30 but where the loadings are ≥ .10 different in absolute magnitude are not considered bivocal, but all loadings ≥ .30 are reported in table. Bivocal items are in bold font. Nonvocal items are in italic font.

**Table 7:** Correlations between factors for 4-factor PAF solution.

	Detachment	Deliberative coping	Emotional coping	Wishful coping
Detachment	1	—	—	—
Deliberative coping	.1349	1	—	—
Emotional coping	.3338	.2156	1	—
Wishful coping	.2084	−.0027	.1724	1
